# A Phage Receptor-Binding Protein as a Promising Tool for the Detection of *Escherichia coli* in Human Specimens

**DOI:** 10.3389/fmicb.2022.871855

**Published:** 2022-06-01

**Authors:** Susana P. Costa, Alexandra P. Cunha, Paulo P. Freitas, Carla M. Carvalho

**Affiliations:** ^1^Centre of Biological Engineering, University of Minho, Braga, Portugal; ^2^LABBELS –Associate Laboratory, Braga/Guimarães, Portugal; ^3^International Iberian Nanotechnology Laboratory, Braga, Portugal; ^4^Instituto de Engenharia de Sistemas e Computadores – Microsistemas e Nanotecnologias and IN – Institute of Nanoscience and Nanotechnology, Lisbon, Portugal

**Keywords:** healthcare-associated infections, cell viability states, diagnostic method, human biological samples, receptor-binding proteins, *Escherichia coli*

## Abstract

*Escherichia coli* is a problematic pathogen that causes life-threatening diseases, being a frequent causative agent of several nosocomial infections such as urinary tract and bloodstream infections. Proper and rapid bacterial identification is critical for allowing prompt and targeted antimicrobial therapy. (Bacterio)phage receptor-binding proteins (RBPs) display high specificity for bacterial surface epitopes and, therefore, are particularly attractive as biorecognition elements, potentially conferring high sensitivity and specificity in bacterial detection. In this study, we elucidated, for the first time, the potential of a recombinant RBP (Gp17) to recognize *E. coli* at different viability states, such as viable but not culturable cells, which are not detected by conventional techniques. Moreover, by using a diagnostic method in which we combined magnetic and spectrofluorimetric approaches, we demonstrated the ability of Gp17 to specifically detect *E. coli* in various human specimens (e.g., whole blood, feces, urine, and saliva) in about 1.5 h, without requiring complex sample processing.

**Graphical Abstract:**
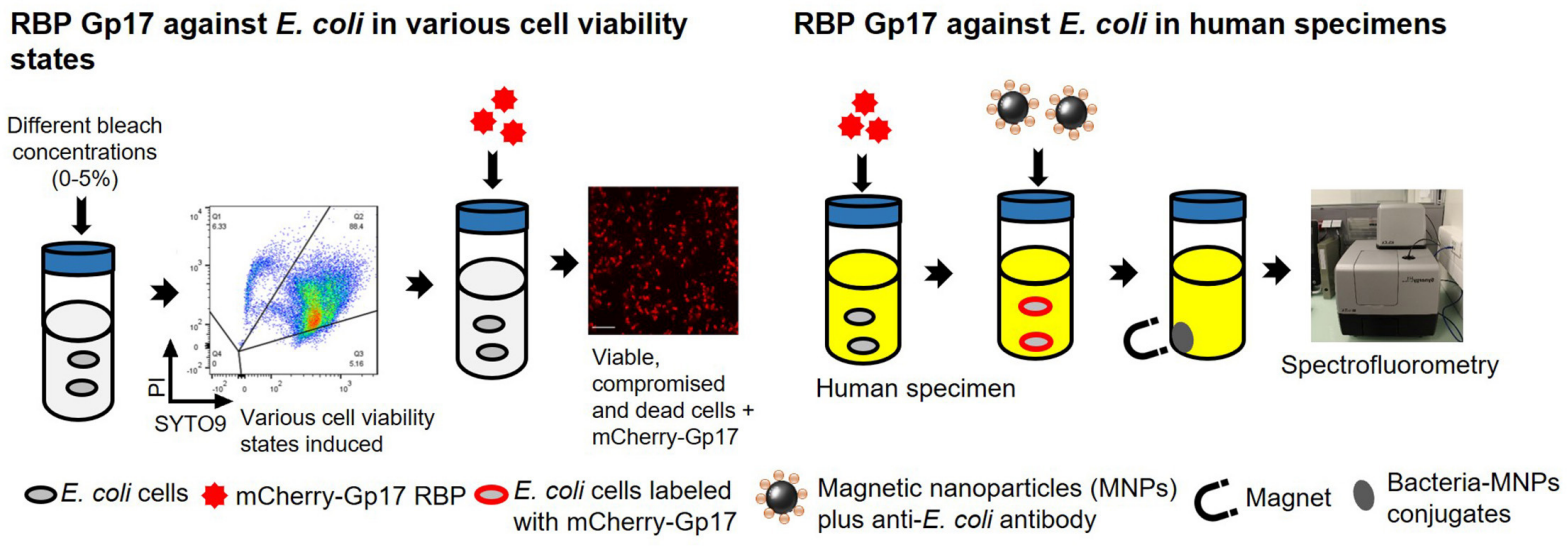
Overview of the experimental studies. The RBP mCherry-Gp17 was tested against *E. coli* at different viability states and used as a recognition molecule for the detection of *E. coli* in human specimens.

## Introduction

Bacterial infections are among the primary causes of death worldwide, and bacterial agents are mainly responsible for healthcare-associated infections (HCAI) that are acquired in hospital environments or other healthcare facilities (World Health Organization, 2011; Haque et al., 2018). These infections arise more than 48 h after hospital admission or within 30 days after receiving medical treatment and have been a worrisome issue (World Health Organization, 2011; Cassini et al., 2016). *Escherichia coli* is an important pathogen responsible for several life-threatening diseases such as pneumonia, meningitis, sepsis, enteric/diarrheal diseases, and urinary tract infections (UTIs) (Kaper et al., 2004). It is the most frequent Gram-negative bacterium isolated from bloodstream infections (BSIs) and the principal causative agent of UTIs (ECDC, 2019).

For effectively controlling these diseases and avoiding the subsequent serious complications, the early diagnosis is of great importance, and it also contributes to the prompt targeted antimicrobial therapy, helps decrease the medical and financial burden, and prevents the spread of antimicrobial resistance (Bereket et al., 2012). The gold standard methods for detecting bacteria are mainly based on culture and colony counting and biochemical tests for the identification of microorganisms (Laupland and Valiquette, 2013; Abayasekara et al., 2017). However, these methodologies are laborious, can be affected by non-microbial material, and require lengthy enrichment steps as a result of the minimal concentration of pathogens in samples and their fastidious growth (Váradi et al., 2017; Rajapaksha et al., 2019). Moreover, bacteria are reported to enter a state, known as viable but non-culturable (VBNC), where they remain metabolically active, keeping their virulence, but cannot grow in standard solid culture media. This compromised state has been described for several pathogenic bacteria and arises due to the influence of different physiological stress conditions (Oliver, 2005, 2010). The appearance of VBNC bacteria may represent a serious threat to public health (Oliver, 2005; Castellani et al., 2013; Pienaar et al., 2016; Zhao et al., 2017) since these cells are present in samples from clinical environments, water distribution systems, and the food industry, but they cannot be detected by conventional culturing detection methods. Moreover, since antibiotics can act as inducers for the VBNC state (Mason et al., 1995; Rivers and Steck, 2001; Ayrapetyan et al., 2015), these cells can remain present and resistant to these compounds, but when the treatment is stopped, they can resuscitate and regain their virulence and thus lead to chronic recurring infections in patients who were considered cured (Rivers and Steck, 2001; Pasquaroli et al., 2013; Ayrapetyan et al., 2018). Additionally, Wilks et al. (2021) reported the appearance of VBNC *E. coli* in urinary catheters resulting from the biofilm development.

In an effort to overcome the disadvantages of the gold standard methods, other technologies like the enzyme-linked immunosorbent assay (ELISA) (Verma et al., 2013), nucleic acid hybridization (McLoughlin, 2011; Frickmann et al., 2017), matrix-assisted laser desorption ionization-time of flight mass spectrometry (MALDI-TOF-MS) (Singhal et al., 2015), and polymerase chain reaction (PCR) (Mothershed and Whitney, 2006) have emerged. Nonetheless, these methods tend to be expensive, involve several steps, suffer from amplification inhibition or infidelity issues, and require prior knowledge of the target DNA sequence or mass spectrometry profile (Ferreira et al., 2011; Marder et al., 2017; Rajapaksha et al., 2019). Another important concern is the fact that these techniques exhibit some issues when applied to complex matrices (Stevens and Jaykus, 2004). Human biological samples, in particular, fecal specimens or whole blood, are among the most complex samples to analyze and detect bacteria due to the presence of numerous inhibitory compounds that interfere in the analysis, especially in the case of amplification- or immunology-based methods (Holland et al., 2000; Stevens and Jaykus, 2004; Opota et al., 2015), making these approaches not attractive for a real monitoring application.

Magnetic trapping (MT) has been widely used for several years as a sample preparation method due to its attractive features (Cudjoe et al., 1995; Kretzer et al., 2007; Qiu et al., 2009; Yang et al., 2013; Lopes et al., 2016). The basic principle is the use of magnetic nanoparticles (MNPs) functionalized with affinity molecules for the target bacterial cells, such as antibodies, to provide a specific capture and isolation of intact cells directly from a complex sample without the need for centrifugation or chromatography methodologies, which are laborious or require specific equipment (Cudjoe et al., 1995; Stevens and Jaykus, 2004). MT presents advantages over other methods, including the effective separation of target bacteria from competitive microflora, the removal of matrix components and potential inhibitors, and the reduction of the sample volume (Stevens and Jaykus, 2004).

The choice of the biorecognition molecule in a diagnostic assay is of great importance to the specificity and robustness of the method (Stevens and Jaykus, 2004; Singh et al., 2012). Several recognition elements for bacteria have been described, such as antibodies, enzymes, aptamers, proteins, or (bacterio)phages (Singh et al., 2013). Receptor-binding proteins (RBPs) from phages, which are responsible for the binding of the phage to the bacterial surface receptors in the initial step of the phage infection (Singh et al., 2010; Schmelcher and Loessner, 2014), have been appointed as great candidates to replace the traditional recognition elements. RBPs are involved in phage adsorption by specifically binding to receptors on the bacterial surfaces, such as proteins, polysaccharides, lipopolysaccharides (LPS), and carbohydrate moieties, dictating the phage infection spectrum (Leiman et al., 2003; Rakhuba et al., 2010). While common receptors for phages infecting Gram-negative bacteria are LPS, capsular polysaccharides (CPS), or bacterial surface proteins (porins and transport proteins), the most frequent in Gram-positive bacteria are the peptidoglycan, teichoic acids, or exposed polysaccharides (Rakhuba et al., 2010; Latka et al., 2017; Dowah and Clokie, 2018). RBPs offer several benefits over other recognition elements like antibodies, such as comparable or higher specificity, high sensitivity, ease of genetic modification, enabling its fusion with fluorescent reporter tags, low production costs, small size, and high chemical and physical resistance (Barbirz et al., 2009; Singh et al., 2010, 2012; Simpson et al., 2015). Therefore, in the past few years, several successful applications of the RBPs for bacterial detection have been reported in the literature, particularly when combined with biosensors (Tay et al., 2012; Shin and Lim, 2018), spectrophotometry (Kunstmann et al., 2018; Santos et al., 2020), ELISA (Denyes et al., 2017; Górska et al., 2018), or lab-on-chip systems (Cunha et al., 2021).

In this study, we unveiled the potential of a recombinant RBP (Gp17) derived from the T7-like polyvalent phage 285p (Xu et al., 2014) as a recognition probe for *E. coli* cells at different viability states (viable, compromised, dead) by using flow cytometry and fluorescence microscopy. Some studies reported the detection of all these cell states by whole phages but are contradictory (Krueger, 1931; Watanabe, 1976; Oda et al., 2004; Awais et al., 2006; Hu et al., 2012; Tlili et al., 2013; Fernandes et al., 2014). Therefore, it was important to assess the binding capacity of a recombinant RBP to these cells, which, to the best of our knowledge, has never been completely elucidated. In this study, we also leveraged the capacity of Gp17 to specifically recognize and detect *E. coli* in complex human biological samples. For this, we combined the benefits of the MT and spectrofluorometry techniques, enabling the separation and detection of *E. coli* in 1.5 h directly from saliva, urine, feces, and whole blood, without requiring complex sample processing.

## Materials and Methods

### Bacteria and Cultivation Procedures

The clinical strains used to test the binding spectrum (Supplementary Table S1) including the *E. coli* HB104 (used as the target bacteria) and *Staphylococcus aureus* HB22 (used as the negative control) were provided by the Hospital of Braga (Portugal). *E. coli* CECT 515 was acquired from the Spanish Type Culture Collection, University of Valencia. The strains were routinely grown overnight in Tryptic Soy Broth (TSB) (Liofilchem) and Luria Bertani (LB) (Liofilchem) at 37°C under agitation (120 rpm) or in solid plates, obtained by adding 12 g/L of agar (Liofilchem).

### Bioinformatic Analysis

The *gp17* was identified as a gene encoding for a tail fiber protein (Gp17) derived from the 285p T7-like polyvalent bacteriophage belonging to the PYO97_8 phage cocktail (Georgian Eliava Institute of Bacteriophage, Microbiology and Virology) (Xu et al., 2014; Villarroel et al., 2017). The protein sequence was analyzed using the Basic Local Alignment Search Tool Protein (BLASTp) non-redundant protein sequence database and expected functional domains were found through Pfam (Finn et al., 2014) and InterProScan (Jones et al., 2014) using the default parameters of the programs. To determine the molecular weight and isoelectric point, the Compute pI/Mw program ExPASy (Artimo et al., 2012) was employed.

### Synthesis, Expression, and Purification of RBP Gp17

The gene *gp17* encoding a potential RBP was synthesized, fused with the *mCherry* gene derived from *Discosoma* sp. at the N-terminus, and cloned into the expression vector pHTP1 (NZYTech) containing a poly-histidine (6xHis) sequence tag at N-terminus. The vector containing the red fluorescent protein mCherry-Gp17 was chemically transformed into *E. coli* BL21 (DE3) cells (Invitrogen). Expression of the fusion protein was carried out as previously described (Costa et al., 2020). In brief, *E. coli* BL21 cells carrying the recombinant plasmid were cultivated at 37°C in an LB medium, adding 50 μg/μl of kanamycin until an optical density (OD) at 600 nm (OD_600 nm_) of 0.6 was reached. The induction of protein overexpression was achieved by adding 1 mM isopropyl-β-D-thiogalactopyranoside (Sigma-Aldrich) and subsequent overnight incubation at 16°C, 120 rpm. Afterward, to lyse the cells, centrifugation was performed (9,000 × *g*, 10 min, 4°C), phosphate lysis buffer (20 mM sodium dihydrogen phosphate, 500 mM sodium chloride, pH 7.4) was used for cell resuspension, and three freeze-thaw cycles and sonication at 12% amplitude (Disintegrator Ultrasonic Mod. 450, Branson) for 15 min (10 s ON and 10 s OFF) were conducted. Then centrifugation (9,000 × *g*, 15 min, 4°C) was carried out to collect the supernatant enriched with soluble proteins, and the protein was purified using a nickel-nitrilotriacetic acid (Ni-NTA) column (Thermo Fisher Scientific). After the washing steps, the elution of proteins was completed with 300 mM imidazole, and fractions were analyzed with SDS-PAGE (12% (w/v) acrylamide) after Blue Safe staining (NZYTech). The protein dialysis and concentration were performed with 0.1 M phosphate buffer pH 7.2 (PB) by using centrifugal filters (Amicon Ultra, 0.5 ml MWCO 10 KDa, Merck Millipore) and proteins were stored at 4°C. The BCA Protein Assay Kit (Thermo Fisher Scientific) assisted in the protein concentration assessment.

### Functional Analysis of mCherry-Gp17 by Fluorescence Microscopy

To assess the ability of the mCherry-Gp17 to bind *E. coli* cells, fluorescence microscopy assays were conducted against *E. coli* HB104, *Klebsiella pneumoniae* HB11, and *S. aureus* HB22 as the negative controls, following the procedure previously described with minor modifications (Costa et al., 2020). In brief, overnight bacterial cells grown in TSB at 37°C were centrifuged (6,000 × *g*, 10 min) and the OD_600 nm_ adjusted to 0.5. Then 400 μl of cultures were centrifuged at 9,000 × *g* for 5 min and resuspended in 40 μl of PB. Then, 20 μl of mCherry-Gp17 at a final concentration of 20 μM was added and incubated at room temperature (RT) for 30 min. Cells were centrifuged at equal conditions, and two washes were done with PB to remove unbound protein and resuspended in 10 μl of PB. Samples were observed at the confocal microscope LSM780 (Zeiss) equipped with a 5 mW 488–645 nm light source in brightfield or under a laser source at 561 nm (DPSS 561-10) for mCherry excitation and setting a bandpass filter (604–735 nm) in the Zeiss ZEN 2010 software for visualization of the protein emission. Control samples of bacterial cells without the addition of the recombinant protein were prepared simultaneously. mCherry alone was used as a negative control.

### Spectrofluorimetric Analysis

Suspensions of clinical strains, which are listed in Supplementary Table S1, were prepared as described in the previous section. The spectrofluorimetric analysis was accomplished according to the protocol described elsewhere (Santos et al., 2020). Suspensions of bacteria (400 μl) were centrifuged at 9,000 × *g* for 5 min and resuspended in 40 μl of PB. Then, 20 μl of mCherry-Gp17 at a final concentration of 5 μM was added and incubated at RT for 30 min. Cells were washed twice by centrifugation (9,000 × *g* for 5 min) using PB to eliminate protein debris and resuspended in 100 μl of PB. The samples were transferred to a black 96-well microplate and examined at a BioTek™ Synergy H1 Hybrid Multi-Mode Microplate Reader with the BioTek Gen5 software. Excitation/emission wavelengths were defined as 570/610 nm (gain 100), and the fluorescence intensity was displayed in arbitrary units (a.u.).

### Flow-Cytometry Assays

#### Optimization of the Protocol to Induce *E. coli* Cells to Different Viability States

Bacterial suspensions of *E. coli* HB104 (used as the target) and of the negative control bacteria were prepared as described in the functional analysis section. Bacterial cells were subjected to different concentrations of sodium hypochlorite (commercial bleach with a stock concentration of 5%) to induce the cells to enter a compromised state (Liu et al., 2009; Fernandes et al., 2014; Ye et al., 2020). Dead cells were prepared by incubation with 5% of bleach (Aranke et al., 2021).

Bacterial suspensions (1 ml) were centrifuged at 7,000 × *g* for 10 min and resuspended in 1 ml of each of the several concentrations of commercial bleach used, which were prepared in PB: 0.000, 0.006, 0.007, 0.020, 0.030, 0.050, and 5.000% (v/v). Samples were incubated for 10 min, then centrifuged at 7,000 × *g* for 10 min at 4°C and washed twice with PB. The culturable cells were quantified after each treatment through colony forming units (CFU) counting.

#### Assessment of Cell Viability

The viability of the cells was estimated after subjecting bacteria to the aforementioned bleach concentrations and staining the cells with the LIVE/DEAD BacLight Bacterial Viability and Counting Kit (Molecular Probes) according to the manufacturer's instructions (Berney et al., 2007; Singh et al., 2012). Later, cells were examined either by confocal microscopy (Zeiss LSM780) or by flow cytometry (Bio-Rad S3e Cell Sorter). In the flow cytometry analysis, the gating strategy was defined according to the LIVE/DEAD BacLight kit (Berney et al., 2007). Microspheres of 6 μm diameter were used as the standard for absolute cell quantification. The results obtained in flow cytometry were analyzed using the FlowJo software (Tree Star, Ashland, OR).

#### Labeling of Cells With mCherry-Gp17 and Flow Cytometry Analysis

The labeling of cells in different viability states with mCherry-Gp17 was carried out as described above for the spectrofluorimetric analysis. The samples were analyzed by flow cytometry using a Bio-Rad S3e Cell Sorter equipped with a dual laser (488/561 nm) and a 615/25 nm filter, allowing the mCherry fluorescence to be detected on the FL3 channel. According to our previous work (Costa et al., 2020), a total of 45,000 events were acquired with a sample flow rate of 10 μl/min. Data analysis was performed using FLOWJO-Single Cell Analysis Software v10 (BD, New Jersey, USA).

### Spectrofluorimetric Magnetic Sandwich Assay

The spectrofluorimetric magnetic sandwich assay was set up starting with the incubation of cells with the mCherry-Gp17 for 30 min and then magnetically labeled with antibody-functionalized MNPs.

#### Preparation of the Functionalized MNPs

To prepare the MNPs, 50 μl of commercial 250 nm streptavidin-coated MNPs (Nanomag-D, Micromod, 4.9 × 10^11^ particles/ml) were rinsed twice with 500 μl of 0.1 M PB Tween 20 (0.05%, v/v) by removing the supernatant with a magnetic concentrator (Dynal-Biotech). Then, 500 μl of the biotinylated *E. coli* polyclonal antibody [LSBio (LifeSpan) Cat# LS-C56164-1, RRID:AB_1509874] at a final concentration of 50 μg/ml were mixed with the MNPs and left on an orbital shaker at 500 rpm, 20°C for 2 h of incubation. Following functionalization, the same process was employed to withdraw the supernatant containing the unbound antibody. The MNPs blocking was carried out by adding 500 μl of bovine serum albumin (BSA) at 5% (w/v) in PB and incubating for 1 h under equal conditions. After supernatant removal, the antibody-conjugated MNPs were washed with PB Tween, resuspended in 50 μl, and kept at 4°C as stock.

#### Magnetic Sandwich Assay

The magnetic sandwich assay was first performed in buffer and in defibrinated horse blood (Probiológica), with cultures of *E. coli* HB104 (used as the target) and *S. aureus* HB22 (used as the negative control) that were prepared as aforementioned. Control samples without any bacteria added were prepared at the same time and submitted to the same process. The bacterial suspensions (400 μl) in PB or horse blood were centrifuged at 9,000 × *g* for 5 min and resuspended in 40 μl of PB. Then, 20 μl of mCherry-Gp17 at a final concentration of 5 μM was added and incubated at RT for 30 min. Then, 4 μl of the antibody-conjugated MNPs stock was added to the samples, completing the volume to 400 μl with PB. The MNPs and bacteria were incubated for 1 h in an orbital shaker at 500 rpm, 20°C. After, the supernatants were gently removed using the magnetic concentrator, and the MNPs were washed twice with 400 μl of PB Tween. Both supernatants and washes were stored for CFUs assessment. Finally, the MNPs were resuspended in 100 μl of PB and immediately analyzed on the spectrofluorometer according to the settings described above. The bacterial capture efficiency was calculated using the following equation:


(1) $$\begin{array}{l} \;Bacterial\;capture\;efficiency\;\left(\%\right)\\ =\frac{CFUs\;I-\left(CFUs\;S+CFUs\;W\right)}{CFUs\;I}\times 100 \end{array}$$

Here, *CFUs I* represent the CFUs initially added, and the *CFUs S* and *CFUs W* represent the CFUs from supernatants and washes, respectively.

#### Detection of Bacteria in Different Human Biological Specimens

All human biological samples used in this study were collected from healthy adult volunteers upon written informed consent. The same type of biological samples were mixed and thus processed de-identified in this study, being the data and samples fully anonymized. Samples of saliva, feces, and urine were collected using a plastic tube. Whole blood was collected using EDTA blood collection tubes (BD Vacutainer). After collection, fecal samples were filtered. Bacterial suspensions (*E. coli* HB104 as target and *S. aureus* HB22 as negative control) were prepared as described before, and 1 ml of each suspension was centrifuged (9,000 × *g*, 5 min) and resuspended in 1 ml of each human specimen. Then, the magnetic sandwich assay was performed as described in the previous section. Bacteria in buffer and control samples without any bacteria added were prepared at the same time and submitted to the same process.

### Statistical Analysis

All data are represented as mean ± SD (standard deviation). For **Figures 4, 5**, multiple comparisons of means were performed using two-way ANOVA followed by Sidak's multiple comparison test (*p*-value < 0.0001) and Dunnett's multiple comparison test (*p*-value < 0.0001), respectively.

## Results

### Bioinformatics Analysis

Phage RBPs are regularly related to tail fiber proteins, tail spikes, or spike proteins (Simpson et al., 2016). The gene *gp17* was identified as encoding a tail fiber protein in the *E. coli* phage 285p from a pyophage (PYO) cocktail (Xu et al., 2014; Villarroel et al., 2017). To further confirm, the sequence of the encoded protein (Gp17) was compared in terms of homology with other phage tail proteins deposited at the National Center for Biotechnology Information (NCBI) database. The results indicate that Gp17 has homology with tail fiber proteins from other *E. coli* phages such as phage BA14 (72% homology), phage PhiV-1 (71%), or phage P483 (60%). Also, the Gp17 has homology with tails from phages infecting *Enterobacter* (*Enterobacter* phage PZJ0206 tail, 95%), *Salmonella* (*Salmonella* phage BSP161 tail, 88%), *Yersinia* (*Yersinia* phage PYPS50 tail, 87%), and *Erwinia* (*Erwinia* phage FE44 tail, 73%). The search for domains and families found hits with the phage_T7_tail_fiber family at N-terminus.

### Functional Analysis of mCherry-Gp17

**Figure 1:**
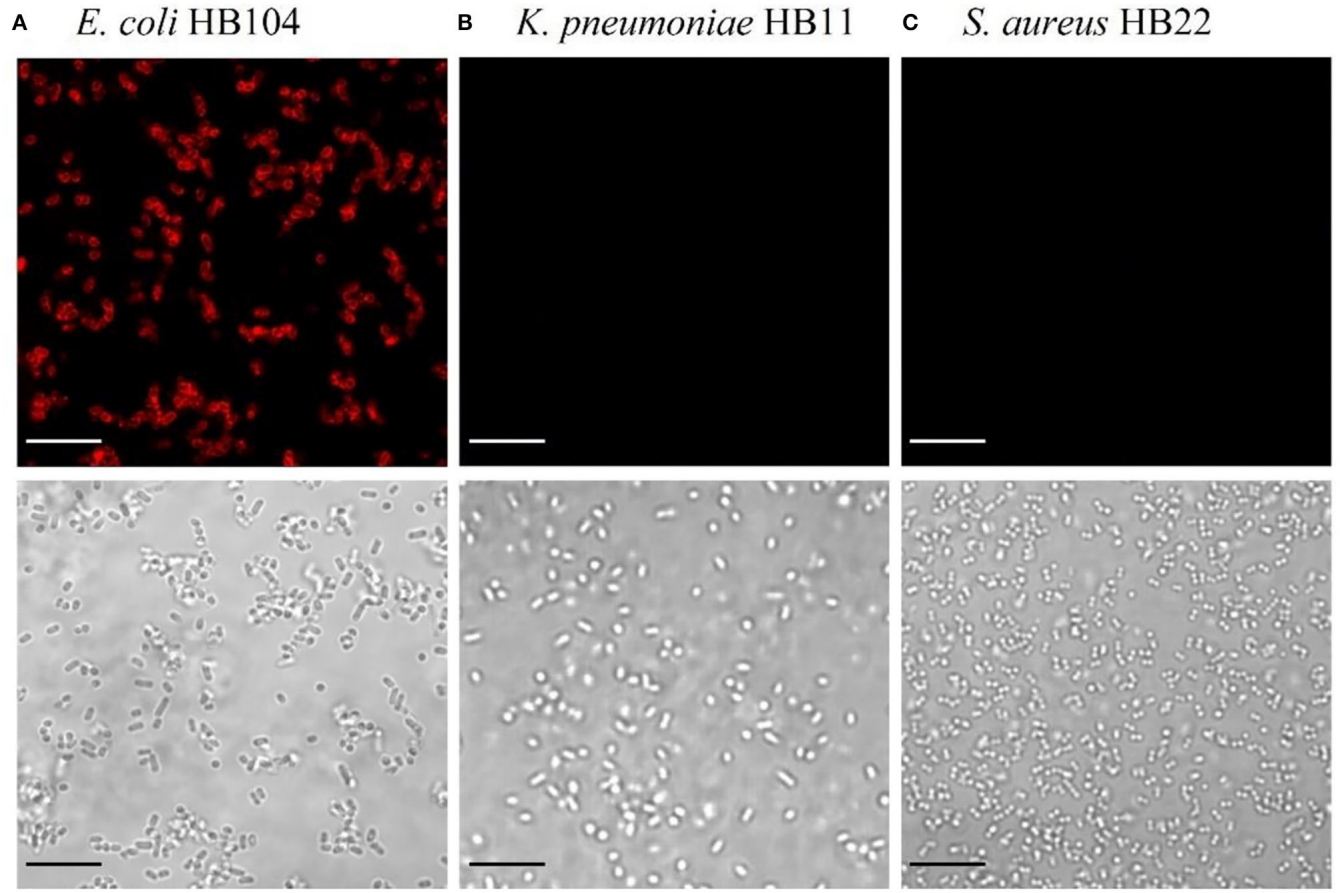
Functional analysis of the RBP mCherry-Gp17. Fluorescence microscopy images after incubation of mCherry-Gp17 with bacterial cells of the target *E. coli* HB104 **(A)** and the non-target *K. pneumoniae* HB11 **(B)** and *S. aureus* HB22 **(C)**. The scale bar represents 10 μm.

After the identification of the Gp17 as a potential RBP, the protein was synthesized, expressed in *E. coli*, and purified using Ni-NTA columns (Supplementary Figure S1). The functional analysis of this protein was performed to confirm its recognition binding ability to the target bacteria and other bacterial species. This was assessed by fluorescence microscopy through the observation of cells emitting red fluorescence since Gp17 was fused with the reporter protein, mCherry (mCherry-Gp17). As shown in Figure 1, this protein can effectively bind to *E. coli* HB104 cells (Figure 1A) and not to the non-target bacteria *K. pneumoniae* HB11 (Figure 1B) or *S. aureus* HB22 (Figure 1C).

In order to evaluate the binding spectrum of mCherry-Gp17 against a panel of strains of *E. coli* and from other genera, spectrofluorimetric analysis was carried out after incubation of cells with mCherry-Gp17. The results are represented in Supplementary Table S1 and indicate that mCherry-Gp17 was able to bind to 59% of the *E. coli* strains tested without showing unspecific binding to strains belonging to other genera (such as *S. aureus, Enterobacter aerogenes, Pseudomonas aeruginosa*, or *K. pneumoniae*). Most of these strains presented a fluorescent signal ratio of 0.4–0.6, thus the clinical isolate *E. coli* HB104 was selected as the representative strain for further assays.

### Assessment of the Binding Ability of mCherry-Gp17 to Bacterial Cells in Different Viability States

To assess if the protein mCherry-Gp17 could recognize different cell viability states, an assay was first developed and optimized to induce the cells to enter these states (e.g., live, compromised, dead) by using different concentrations of sodium hypochlorite (commercial bleach) (see Supplementary Figure S2). The cell viability was determined through flow cytometry and fluorescence microscopy after incubation of cells with the viability dyes SYTO9 and propidium iodide (PI), together with the CFU validation. These assays permitted to conclude that the treatment with 0.03% and 5% of bleach was the best to induce most of the cells into the compromised and dead states, respectively (Supplementary Figure S2).

**Figure 2:**
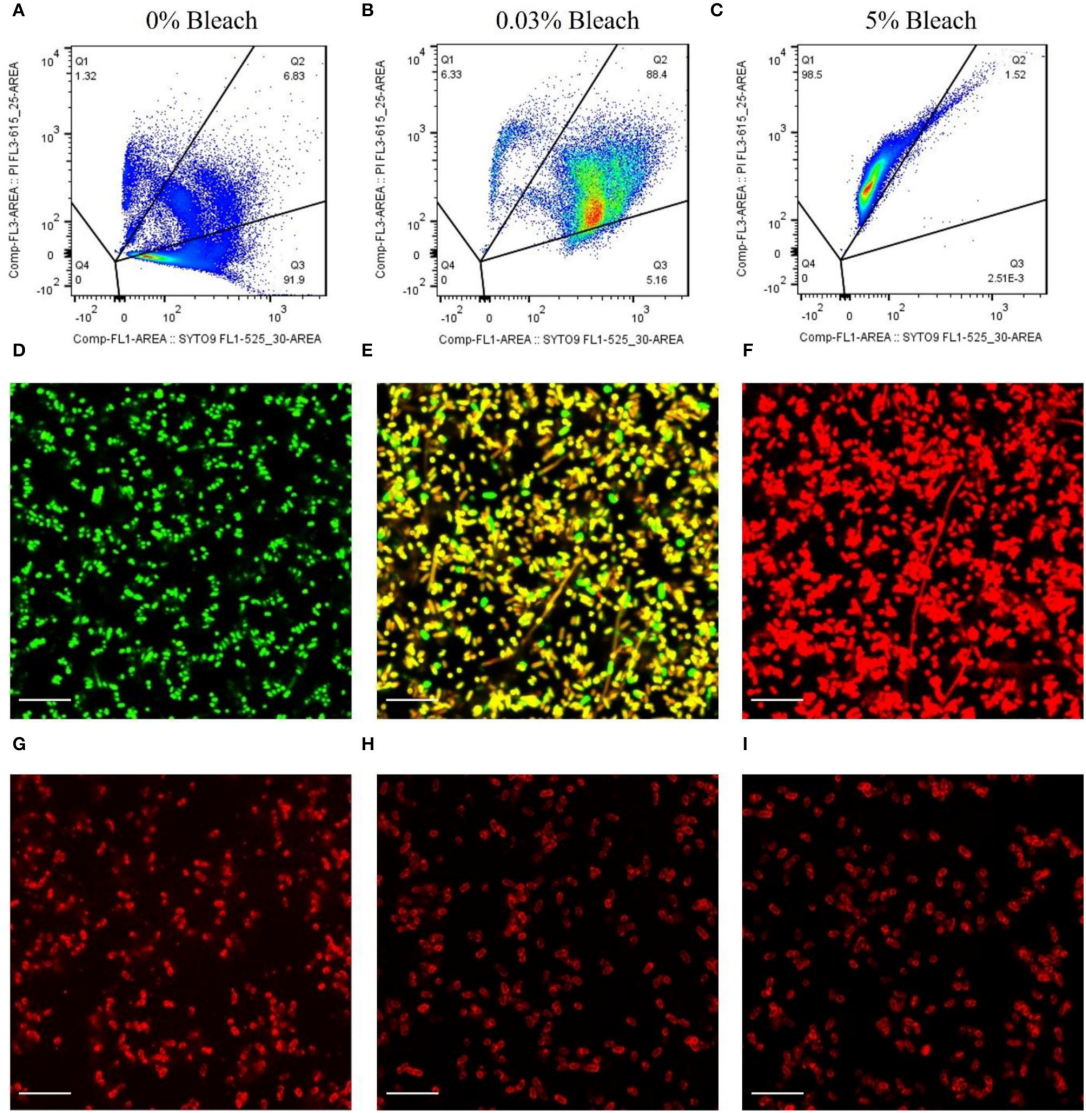
Assessment of the binding ability of mCherry-Gp17 to *E. coli* cells in different viability states. **(A–C)** Representative flow cytometry plots showing the percentage of the dead (Q1), compromised (Q2), and viable (Q3) cells present after treatment with bleach at different concentrations. Cells were labeled with SYTO 9 (green) and PI (red). **(D–F)** Fluorescence microscopy images of *E. coli* cells treated with 0% bleach (viable), 0.03% bleach (compromised), and 5% bleach (dead) stained with SYTO 9 (green) and PI (red). **(G–I)** Fluorescence microscopy images of cells treated with 0% bleach (viable), 0.03% bleach (compromised), and 5% bleach (dead) after incubation with mCherry-GP17.

Figures 2A–C depicts the representative dot plots showing the percentage of cells in each quadrant, defined according to the gating strategy (Supplementary Material) and for the different treatments that were submitted: 0% of bleach (Figure 2A), 0.03% (Figure 2B), and 5% (Figure 2C). These cells were observed by fluorescence microscopy, and the results corroborate the flow cytometry assays. The cells that were not subjected to any treatment appeared green (Figure 2D), and most of the cells (≈92%, Figure 2A) were considered viable. Also, after bleach treatment, some cells appeared double-labeled (≈90%, Figure 2B), showing intermediate colors from yellow to orange by microscopy (Figure 2E), indicating the presence of compromised cells which on CFU counting revealed to be unable to grow on standard solid media (Supplementary Figure S2), implying that these cells are in the VBNC state (Stiefel et al., 2015; Truchado et al., 2020). Moreover, cells submitted to 5% of bleach appeared as dead bacteria cells by flow cytometry (≈99%, Figure 2C), emitting red fluorescence (Figure 2F).

**Figure 3:**
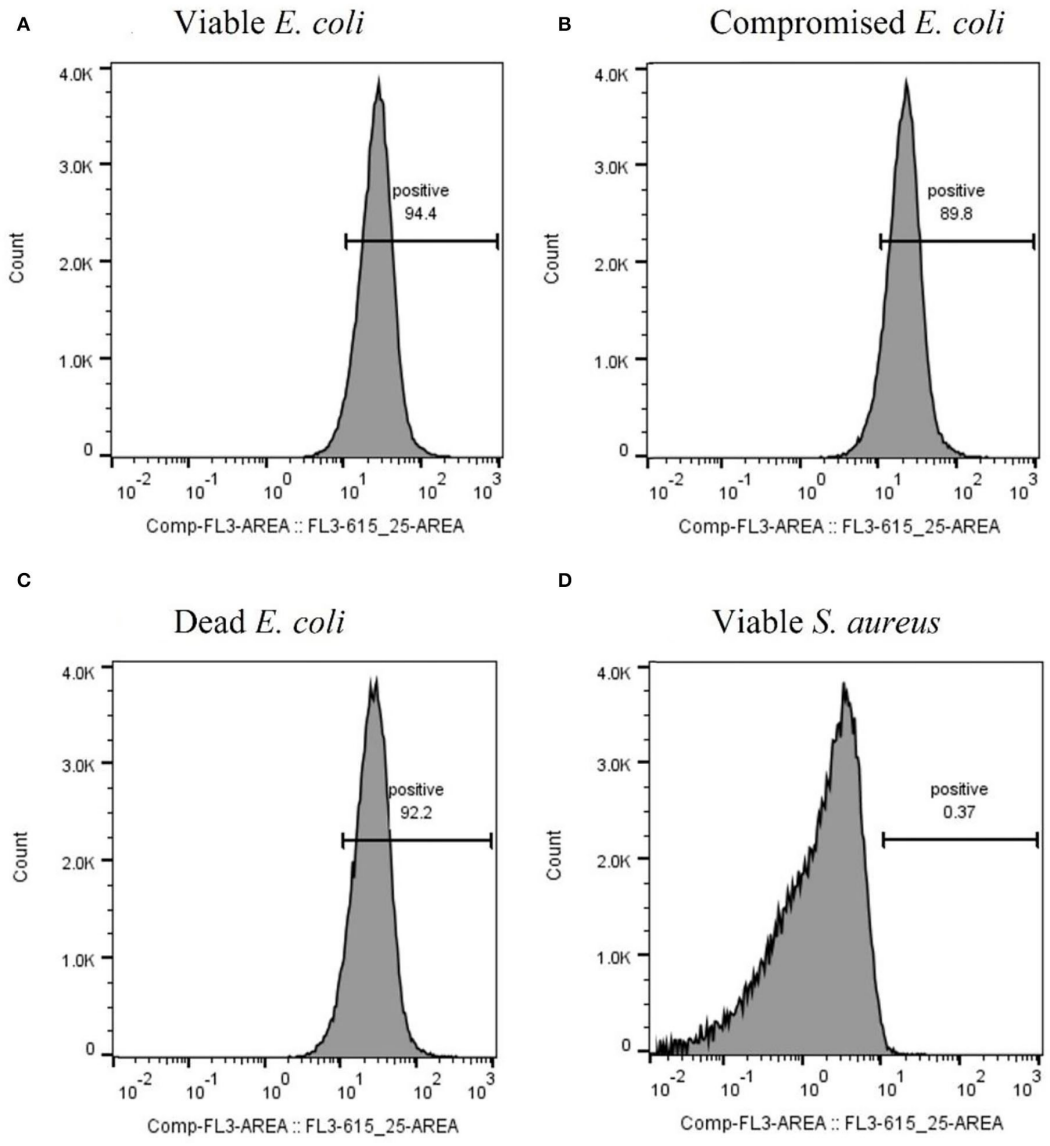
Assessment of the binding ability of mCherry-Gp17 to cells in different viability states by flow cytometry. Representative histograms were obtained from the analysis of viable **(A)**, compromised **(B)**, and dead **(C)** *E. coli* HB104 cells and viable *S. aureus* HB22 **(D)** (negative control) after incubation with mCherry-Gp17.

After optimizing the procedure for converting the cells into compromised (such as VBNC) and dead cells, the ability of the RBP mCherry-Gp17 to recognize and bind to cells at different viability states was studied (Figures 2G–I, 3). The results revealed that the protein was able to recognize viable (Figures 2G, 3A), compromised (Figures 2H, 3B), and dead cells (Figures 2I, 3C). The percentage of positive events was similar between the different types of samples (≈90%) (Figures 3A–C), suggesting that the RBPs recognized the cells at different viability states in the same manner. In the case of the negative control, which corresponds to *S. aureus* HB22 cells labeled with mCherry-Gp17 (non-target bacteria, Figure 3D), just a neglected number of cells were recorded as positive (0.37%).

### Spectrofluorimetric Magnetic Sandwich Assay for *E. coli* Detection in Spiked Human Specimens

Since HCAIs can be presented in different sites of the body (Haque et al., 2018), it is important to assess the ability of the RBP to detect pathogens in different types of human biological specimens. After obtaining the promising results from the preliminary experiments performed in buffer and horse blood (Supplementary Figure S3), samples of urine, blood, feces, and saliva provided by healthy volunteers were spiked with *E. coli* HB104 cells or with *S. aureus* HB22 as the negative control.

The samples were first incubated with mCherry-Gp17 and then cells were magnetically separated using MNPs functionalized with an anti-*E. coli* antibody, and the resulting fluorescent signals were measured on the spectrofluorometer.

**Figure 4:**
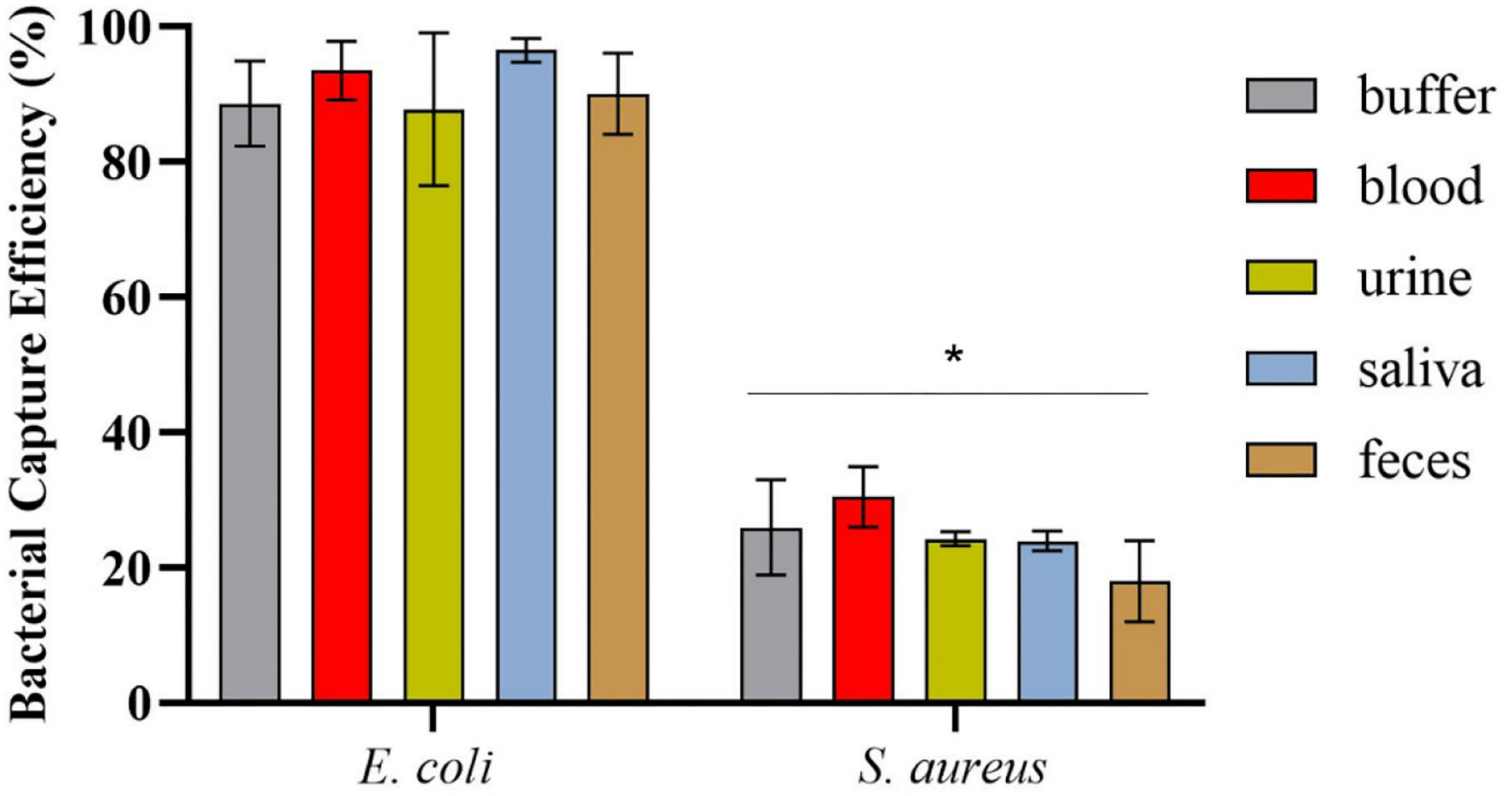
Bacterial capture efficiencies (in percentage) obtained in the magnetic sandwich assay performed in the different types of human specimens for *E. coli* HB104 (target bacteria) and *S. aureus* HB22 (non-target bacteria), assessed by CFU counting. Errors bars represent the standard deviation of the average of three independent experiments (*n* = 3). *Statistical analysis was performed comparing the percentage of bacterial capture obtained for the target bacteria *E. coli* and the negative control *S. aureus* in each of the human specimens. Multiple comparisons were done using the two-way ANOVA with Sidak's multiple comparison test (*p-*value < 0.0001).

After magnetic enrichment, supernatants and washes were analyzed by CFU counting, and the bacterial capture efficiencies are displayed in Figure 4. The results demonstrated high bacterial capture efficiency (more than 87%) for *E. coli* HB104 present in different human biological samples, proving the efficacy of this methodology to recover bacterial cells from complex sample matrices. Moreover, although some unspecific capture occurred for the negative control with *S. aureus*, especially in whole blood (30% of capture efficiency), these results were significantly different (*p*-value < 0.0001) from the values obtained for *E. coli*.

**Figure 5:**
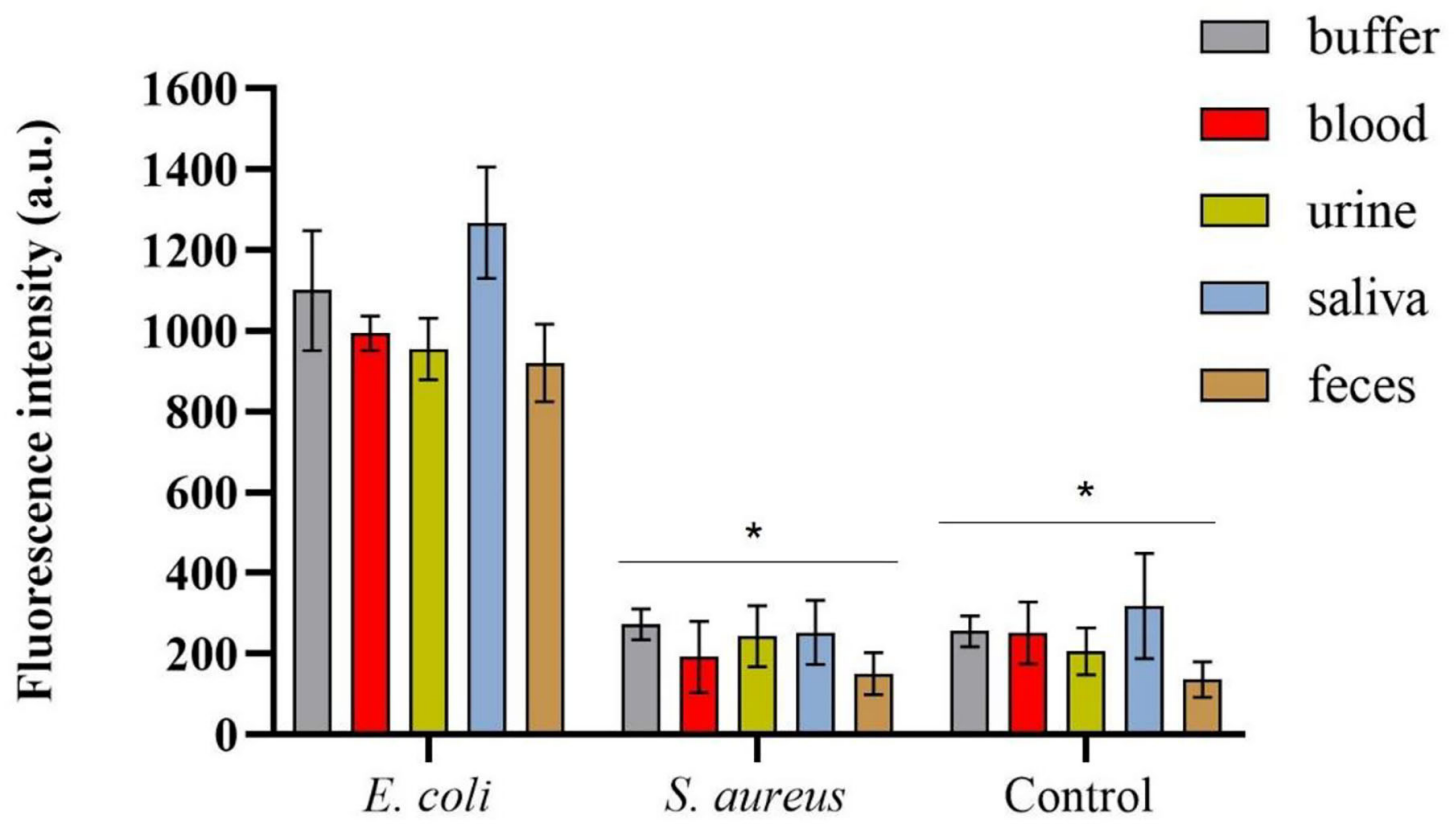
Spectrofluorimetric results of the magnetic sandwich assay performed in the different types of human specimens for *E. coli* HB104 (used as target bacteria), *S. aureus* HB22 (used as non-target bacteria), and for the control without bacteria added. Errors bars represent the standard deviation of the average of three measurements in the three independent assays (*n* = 3). *Statistical analysis was done comparing the signals in each of the human specimens obtained for *S. aureus* and control (without bacteria) with the signal of *E. coli* (target bacteria), defined as “positive” control, in the same samples. Multiple comparisons were done using the two-way ANOVA test (*p*-value < 0.0001) followed by Dunnett's multiple comparison test (*p*-value < 0.0001). a.u. stands for arbitrary units.

The spectrofluorimetric results, which correspond to the fluorescence signals derived from the conjugated mCherry-Gp17-bacteria-MNPs that remained after the washing steps, are shown in Figure 5. It was possible to observe a statistically significant difference (*p*-value < 0.0001) between the fluorescence signals acquired for *E. coli* HB104 in the different human biological samples (fluorescent signals over 900 a.u.) and the negative controls, namely the non-target *S. aureus* HB22 and the samples without bacteria that were subjected to the same process (means of fluorescence less than 300 a.u.) (Figure 5). Also, in the fluorescence microscopy analysis of conjugated mCherry-Gp17-bacteria-MNPs obtained in an assay performed in the blood (Supplementary Figure S4), it was not possible to see any fluorescence signal for the negative control bacteria *S. aureus* HB22, which is visible in the case of *E. coli* HB104 cells.

## Discussion

Considering that *E. coli* is among the most common microorganisms isolated from HCAIs, particularly being an important pathogen responsible for BSIs and UTIs (ECDC, 2019), we have assessed the potential of a phage RBP as a recognition probe for *E. coli* detection in different human biological samples. Accordingly, after identifying Gp17 as a potential RBP, this protein was synthesized and fused with mCherry as a reporter protein. The binding ability of this protein was tested first by fluorescence microscopy and then by spectrofluorometry against a larger panel of clinically isolated strains, derived from blood, urine, and skin exudates, among others. The results revealed that Gp17 successfully recognize 60% of *E. coli* strains and did not show affinity against other bacterial species that are also prevalent causative agents of HCAIs (Bassetti et al., 2017; Lee et al., 2018; ECDC, 2019).

Besides the affinity for viable cells, which has been extensively studied for RBPs (Fujinami et al., 2007; Javed et al., 2013; Denyes et al., 2017; He et al., 2018; Santos et al., 2020), we have tested the binding capacity of the recombinant Gp17 against other cell viability states, namely dead and compromised cells, which to the best of our knowledge have never been completely addressed. The results demonstrated that Gp17 can recognize cells in all these cell states, which may indicate that the receptors involved in the RBP's cell recognition and binding remained available independently of the cell viability. T-even phage adsorption is mediated by long-tail fibers, which target and bind to host physiological receptors such as LPS and outer membrane proteins (Drexler et al., 1989; Yoichi et al., 2005; González-García et al., 2015a,b; Lupo et al., 2015), commonly known as the receptors of RBPs (Rakhuba et al., 2010). According to some studies that have focused on the use of whole phages against VBNC and dead cells, this thematic seems to be controversial once some authors have shown that phages attach to dead cells (Krueger, 1931; Watanabe, 1976; Oda et al., 2004; Awais et al., 2006; Hu et al., 2012), in contrast with others (Tlili et al., 2013; Fernandes et al., 2014). The only consensus was the ability of the phages to adsorb to VBNC cells (Oda et al., 2004; Awais et al., 2006; Fernandes et al., 2014). Akusobi et al. (2018) reported that *E. coli* phage PP01 was able to effectively adsorb to dead cells, and this effect was enhanced through natural point mutations occurring on its long tail fiber (gp38). This protein was previously described as responsible for the reversible binding of the phage PP01 to the outer membrane protein C (OmpC) on *E. coli* O157:H7's cell surface (Yoichi et al., 2005).

The ability of Gp17 to detect viable and compromised cells can be considered an advantage for real-time monitoring of bacterial infections. Particularly, the recognition of compromised cells like VBNC is of extreme importance since these cells, which were not detected by the standard culture methods, can “resuscitate” when in favorable conditions and cause diseases (Du et al., 2007; Li et al., 2014). Nonetheless, the use of Gp17 as a recognition molecule should be thoroughly considered once a bacterial pre-enrichment step may be required to overcome the possibility of detection of dead cells, potentially causing false positives (Denyes et al., 2017).

The application of phage RBPs as recognition elements for bacterial detection in human specimens is limited, with only a few studies reporting their use in urine (He et al., 2018; Shi et al., 2021). Therefore, it was important to prove the applicability of Gp17 for the detection of *E. coli* in biological samples and thus evaluate whether these elements can bind bacterial cells even in the presence of many other irrelevant components, known as having interferent or inhibitory activity in other methodologies, such as in PCR-based and immunology techniques (Holland et al., 2000; Stevens and Jaykus, 2004; Opota et al., 2015). To achieve this goal, we used the Gp17 as a probe and combined it with the advantages of MT and spectrofluorometry techniques for bacterial capture and detection, respectively. The use of MT as a sample preparation approach assisted in the efficient isolation of the target bacterial cells (more than 87%) from the human specimens, allowing the cleaning of the interfering components in a fast and simple way, without the need for laborious centrifugation steps. MT has been shown as a promising sample preparation approach in diverse systems (Favrin et al., 2001; Schmelcher et al., 2010; Wang and Alocilja, 2015; Ngamsom et al., 2016; Chen et al., 2020), including some using RBPs to functionalize the MNPs (Kretzer et al., 2007; Denyes et al., 2017; Cunha et al., 2021). The results from the MT also revealed some capture for the non-target bacteria *S. aureus*. This cross-reactivity was expected since MNPs were functionalized with a polyclonal antibody, which is commonly associated with cross-reactivity issues (Frank, 2002). This broad capture was not a problem in the further spectrofluorimetric assays, because the Gp17 only recognized *E. coli*, guaranteeing the specificity of the detection assay. Indeed, the results obtained from these measurements revealed distinguishable signals derived from the detection of *E. coli* (around 1,000 a.u.) in several human biological specimens compared to the signals obtained in the controls (about 300 a.u.). This background signal can be the result of some residual binding occurring between the sample components and the Gp17, or due to remaining components that were not fully washed and that can have auto-fluorescence, such as the red blood cells (Azevedo et al., 2011; Skvarc et al., 2013). Nonetheless, the fluorescence microscopy analysis of the conjugated mCherry-Gp17-bacteria-MNPs revealed no fluorescence for the negative control bacteria *S. aureus* HB22, which is visible in the case of *E. coli* HB104 cells. This suggests that the residual signal obtained in the spectrofluorimetric measurements was not due to the non-specificity of the Gp17 but rather to the sensitivity of the methodology.

Overall, the RBP-based methodology enabled the specific detection of *E. coli* in about 1.5 h directly in human specimens, without the need for complex sample processing steps that are laborious and time-consuming. Although the inherent detection limit of the equipment restricted the application of the methodology in lower bacterial contents (less than ≈ 10^7^ CFU), this can be overcome by using more sensitive analytical methods, like optical sensors (Jin et al., 2017).

The developed method can be adapted for the detection of other bacterial species. The broad capture achieved by the functionalized MNPs may be advantageous once there is no need for another antibody targeting other bacterial species, possibly decreasing the costs associated with the detection assay. Thus, it can be a benefit for multiplex bacterial detection, once other phage proteins, providing the high specificity, can be used to target other bacterial species that may be present at the same time in these human biological samples. Also, since this methodology detects and identifies the specific causative agents without compromising their viability, it enables their use for subsequent antibiotic susceptibility tests. This is an important feature of the designed assay since these tests are not possible to be conducted with many other techniques, for instance, nucleic acid-based, that can cause cell degradation or death. This is essential for choosing the most appropriate antimicrobial treatment and to support an efficient therapy, thereby reducing the overuse and misuse of antibiotics and associated adverse outcomes (Afshari et al., 2012).

Further evaluation of our assay in bacteria in biofilm growth mode (Wilks et al., 2021) and mixed bacterial populations, preferentially in combination with different probes specific to other pathogens, would greatly contribute to a more precise assessment of the feasibility of this method. Moreover, it would be interesting to test the RBP against bacteria submitted to other conditions that affect their viability (Liu et al., 2008; Zhao et al., 2013; Wei and Zhao, 2018) or cells under different physiological states, namely, induced by prolonged substrate limitation (Hadas et al., 1997; Nabergoj et al., 2018).

## Conclusion

In this study, we provided further insights into the ability of an RBP to recognize with high specificity *E. coli* strains at different viability states. Also, when this protein was combined with the outstanding benefits of spectrophotometry and magnetic approaches, it enabled the detection of *E. coli* in different human biological matrices, proving the feasibility of the use of RBPs as exceptional probing elements in biosensing technologies.

## Data Availability Statement

The original contributions presented in the study are included in the article/Supplementary Material, further inquiries can be directed to the corresponding author.

## Ethics Statement

Ethical review and approval was not required for this study in accordance with the local legislation and institutional requirements. The participants provided their written informed consent to participate in this study and the data of the participants were made anonymous in such a way that the data subject can no longer be identified.

## Author Contributions

SC and AC performed the laboratory work. SC, PF, and CC were responsible for the conceptualization of the study. SC analyzed the data and wrote the original draft of the manuscript. SC and CC performed the review and editing of the manuscript. All authors read and approved the manuscript.

## Funding

This study was supported by the Portuguese Foundation for Science and Technology (FCT) under the scope of the project Phages-on-chip PTDC/BTM-SAL/32442/2017 (POCI-01-0145-FEDER-032442) and the strategic funding of the research units CEB (UIDB/04469/2020) and INESC MN (UID/05367/2020) through the pluriannual BASE and PROGRAMATICO financing and BioTecNorte operation (NORTE-01-0145-FEDER-000004) funded by the European Regional Development Fund under the scope of Norte2020, Programa Operacional Regional do Norte. SC was supported by the FCT grant SFRH/BD/130098/2017.

## Conflict of Interest

The authors declare that the research was conducted in the absence of any commercial or financial relationships that could be construed as a potential conflict of interest.

## Publisher's Note

All claims expressed in this article are solely those of the authors and do not necessarily represent those of their affiliated organizations, or those of the publisher, the editors and the reviewers. Any product that may be evaluated in this article, or claim that may be made by its manufacturer, is not guaranteed or endorsed by the publisher.
