# Supplementary material for: A Phage Receptor-Binding Protein as a Promising Tool for the Detection of Escherichia coli in Human Specimens
Source: Front Microbiol. 2022 Jun 1;13:871855. doi: 10.3389/fmicb.2022.871855 (PMC9202026; doi:10.3389/fmicb.2022.871855)
Supplement: Supplementary file 1 [file Data_Sheet_1.pdf]

Supplementary Material

1. Characterization and functional analysis of mCherry-Gp17

Table S1 - List of bacterial strains that were used in this study to assess the binding spectrum and specificity of the RBP mCherry-Gp17 through spectrofluorometry.

Bacterial strain	Origin	Normalized signal
***Escherichia coli* strains**		
E. coli BL21 (Invitrogen)	NA	0.57
E. coli CECT 515	Urine	0.01
E. coli HB2	Blood	0.59
E. coli HB3	Blood	0.01
E. coli HB4	Blood	0.55
E. coli HB5	Blood	0.41
E. coli HB6	Blood	0.01
E. coli HB8	Blood	0.30
E. coli HB9	Blood	0.01
E. coli HB10	Blood	0.02
E. coli HB24	Blood	0.01
E. coli HB25	Blood	0.01
E. coli HB26	Expectoration	0.01
E. coli HB27	Urine	0.44
E. coli HB29	Expectoration	0.58
E. coli HB30	Blood	0.39
E. coli HB38	Blood	0.01
E. coli HB39	Blood	0.01
E. coli HB51	Urine	0.53
E. coli HB60	Blood	0.00
E. coli HB61	Blood	0.55
E coli HB69	Blood	0.02
E. coli HB70	Blood	0.55
E. coli HB71	Urine	0.54
E. coli HB72	Urine	0.62
E. coli HB73	Blood	0.05
E. coli HB74	Urine	0.00
E. coli HB75	Peritoneal fluid	0.01
E. coli HB76	Urine	0.10
E. coli HB77	Urine	0.56
E. coli HB78	Urine	0.13
E. coli HB79	Urine	0.19
E. coli HB80	Urine	0.16
E. coli HB81	Catheter	0.37
E. coli HB82	Urine	0.02
E. coli HB83	Peritoneal fluid	0.39

E. coli HB84	Blood	0.01
E. coli HB85	Urine	0.09
E. coli HB86	Blood	0.22
E. coli HB87	Urine	0.31
E. coli HB88	Urine	0.35
E. coli HB89	Blood	0.19
E. coli HB90	Urine	0.01
E. coli HB91	Urine	0.01
E. coli HB92	Urine	0.53
E. coli HB93	Urine	0.18
E. coli HB94	Urine	0.16
E. coli HB95	Urine	0.16
E. coli HB96	Urine	0.53
E. coli HB97	Urine	0.01
E. coli HB98	Urine	0.21
E. coli HB99	Urine	0.54
E. coli HB100	Urine	0.01
E. coli HB101	Urine	0.41
E. coli HB102	Urine	0.01
E. coli HB103	Urine	0.02
E. coli HB104	Blood	0.55
E. coli HB105	Skin exudate	0.01
E. coli HB106	Skin exudate	1.00
E. coli HB107	Urine	0.02
E. coli HB108	Urine	0.40
**Other bacterial species**		
Pseudomonas aeruginosa HB12	Blood	0.03
Pseudomonas aeruginosa HB79	Blood	0.05
Klebsiella pneumoniae HB11	Blood	0.04
Staphylococcus aureus HB22	Blood	0.05
Enterococcus faecalis HB20	Blood	0.04
Enterobacter aerogenes HB71	Blood	0.03
Enterobacter cloacae HB54	Blood	0.02

Note: The fluorescent signal acquired (in arbitrary units - a.u.) for each bacterial strain when incubated with mCherry-Gp17 was normalized against the higher signal obtained (in this case the signal from *E. coli* HB106). Normalized values <0.06 were considered negative. NA – Not applicable. The values represent the average of three measurements in the two independent assays (n=2).

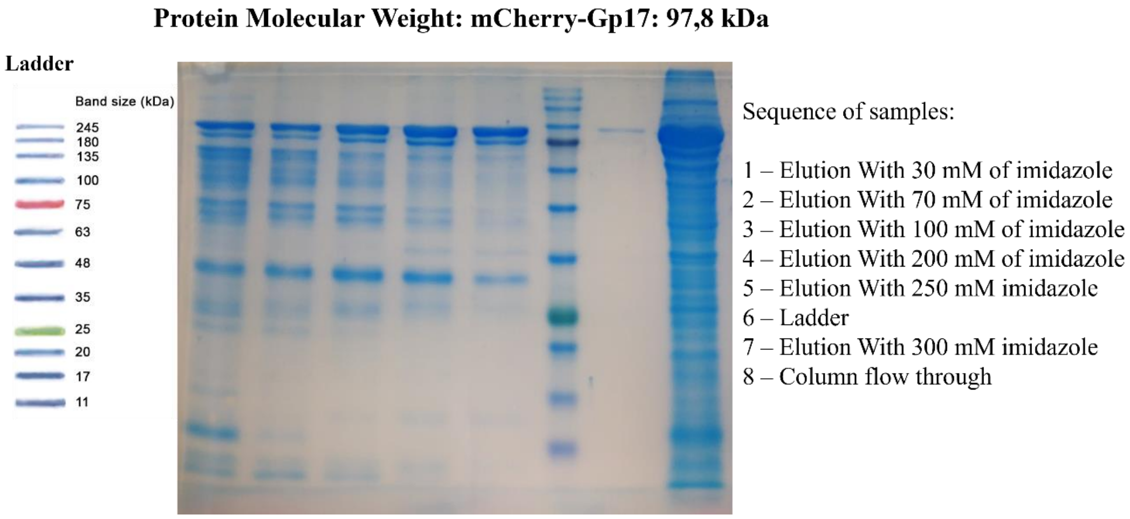

Figure S1 – Representative Sodium Dodecyl sulfate-polyacrylamide gel electrophoresis (SDS-PAGE) of the elution fractions with increased concentrations of imidazole obtained in the mCherry-Gp17 purification procedure using a Ni-NTA column. The Ladder used was the NZYColour Protein Marker II (Nzytech).

2. Optimization of the induction of *E. coli* to different viability states

The induction of cells to different viability states was performed by submitting cells to distinct sodium hypochlorite (commercial bleach) concentrations for a short period. Some reports have mentioned that chlorine or chloramine disinfectants can induce *E. coli* into dead (Aranke et al., 2021) and a viable but nonculturable (VBNC) state (Liu et al., 2009; Ye et al., 2020). The optimization and evaluation of the induction of *E. coli* cells to different viability states were performed by flow-cytometry analysis and fluorescent microscopy, using the SYTO9/PI viability dyes. Also, the CFU counting was performed to assess the viability of cells.

When co-staining with PI and SYTO 9 DNA-binding dyes is performed, generally, SYTO 9 can enter cells independently of their membrane integrity, emitting green fluorescence while PI enters cells with compromised membranes, emitting a red fluorescent signal. Since

PI have a higher affinity to bind DNA than SYTO9, usually dead cells present a red fluorescent signal, and viable (live) cells present green signals (Boulos et al., 1999; Rosenberg et al., 2019).

Five different concentrations of bleach were tested, ranging from 0.006% to 0.05% (v/v) to induce the cells to enter in a compromised state, like VBNC cells. According to the defined flow cytometry gates, it was possible to obtain the percentage of cells in each state and the results are depicted in Figure S2. In Figure S2 are also presented the CFUs obtained in each of the samples. The flow cytometry gates were firstly defined as previously described for the LIVE/DEAD BacLight kit (Berney et al., 2007), in which dead bacterial cells can be visualized in Q1 quadrant and viable cells in Q3 quadrant (Figure 2 A-C). However, in our experiments, for certain concentrations of bleach, it was observed a population that presented yellow to orange color that was defined as compromised cells in the gating strategy adopted in the flow cytometry analysis (double-labeled), as described before (Gião et al., 2009; Fernandes et al., 2014; Truchado et al., 2020), indicating that they are still alive, but their cell membranes can be compromised (Truchado et al., 2020). This double staining cell population can be visualized in the Q2 quadrant (Figure 2 A-C). In our assays, these cells were not able to grow on standard solid media, therefore leading us to believe that these compromised cells are VBNC. The entry of PI has been attributed not only to the damage of membranes but also to the high membrane potential (Kirchhoff and Cypionka, 2017), which indicates that the stain can effectively enter metabolically active cells (Boulos et al., 1999; Gião et al., 2009; Rosenberg et al., 2019). The results for the different bleach treatments indicate that when no treatment was applied, most of the cell population (92%) was stained as green fluorescing cells (Figure S1) and thus considered viable. The concentration of these cells was around 8.5 Log_{10} CFU/mL in all experiments. Conversely, when the cells were treated with 5% of bleach, almost all of the cell

population (99%) was dead which was corroborated by the CFU counting, since cells were unable to grow on media.

The 0.006% bleach concentration, reported as the breakpoint concentration to induce *Salmonella* VBNC cells (Fernandes et al., 2014), was not able to induce the entire *E. coli* cell population to this state. Just a minimal percentage of cells (about 5%) were in the compromised state (Figure S1). The same occurred for the concentration of 0.007%, in which although the percentage of compromised cells slightly increased (12%), it was not able to induce most of the cell population into the compromised state. Then higher bleach concentrations (0.02, 0.03 and 0.05%) were tested. Using a bleach concentration of 0.02% the percentage of compromised cells was 83% and only 11% was considered viable. The CFU counts revealed a decrease in about 2 orders of magnitude relative to the viable cells (in which the initial cell concentration was higher than 8 Log_{10} CFU/mL), only corresponding to the percentage of cells considered as viable (11%), suggesting that the remaining population, defined as compromised, lost the ability to grow on standard media, potentially being VBNC cells.

The concentration of 0.03% was defined as the breakpoint, where the highest percentage of compromised cells was obtained (about 90%), showing a decrease in CFUs of about 3 orders of magnitude, only corresponding to the percentage of viable cells (4%) able to grow in standard solid media. The compromised cells (90%), not detected on solid media are potential VBNC. At 0.05% of bleach, the percentage of compromised cells decreased (to 73%), and the same occurred for the viable cells (0.3%) and the dead cells increased (to 27%).

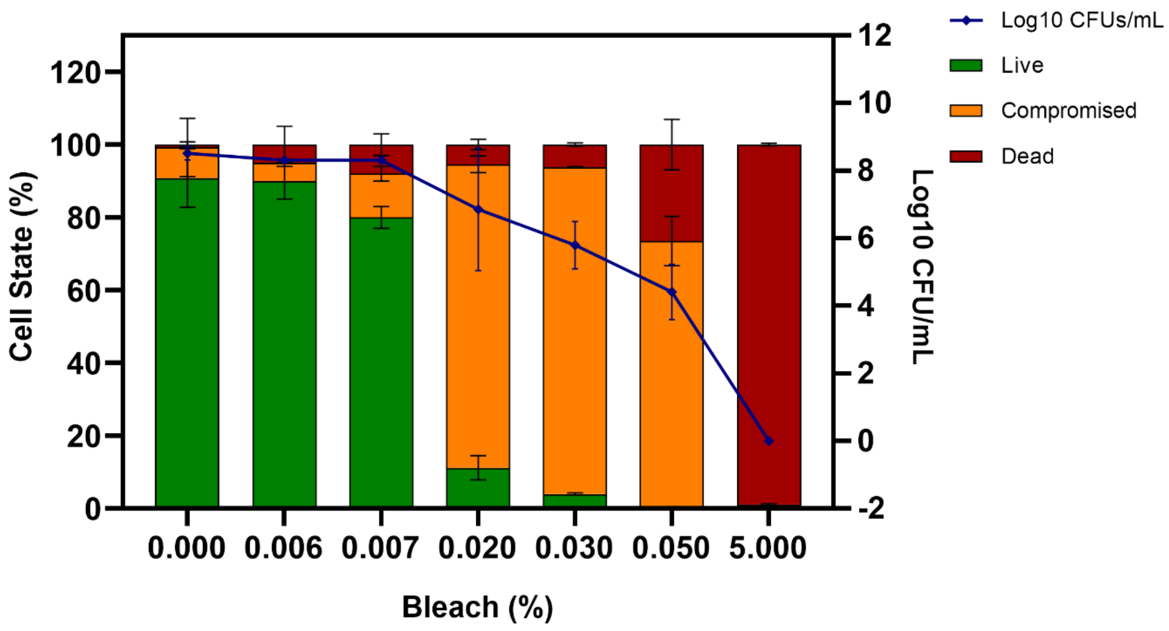

Figure S2 - Assessment of the viability state of *E. coli* cells after treatment with different concentrations of bleach by two different evaluation methods, CFU counts on standard solid culture media (blue line), and flow cytometry results of the relative percentage of cells in the viable (green bars), compromised (orange bars) and dead (red bars) states. Cells were stained with SYTO9 and PI. Errors bars represent the standard deviation of the average of three measurements in the three independent assays (n=3).

3. Spectrofluorimetric magnetic sandwich assay for *E. coli* detection in spiked human specimens

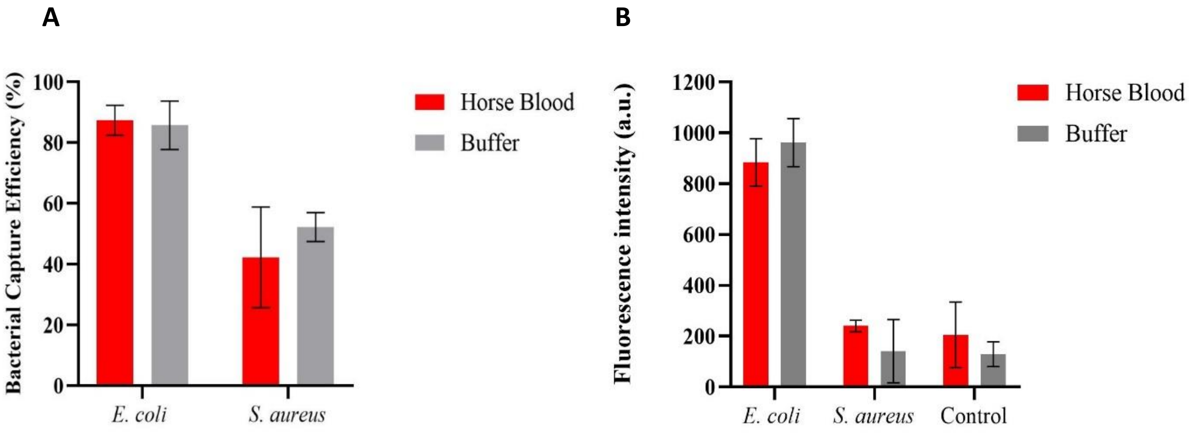

Figure S3 - Results from the spectrofluorometric magnetic sandwich assay performed on the horse blood and buffer in the preliminary experiments. (A) Results from the magnetic capture efficiency in percentage, assessed by CFU counting, for the host bacteria *E. coli* HB104 and non-target bacteria, *S. aureus* HB22. Errors bars represent the standard deviation of the average of three independent assays (n=3). (B) Spectrofluorometric results after the magnetic sandwich assay for the *E. coli* HB104 as host, *S. aureus* HB22, non-target bacteria, and for the control, without bacteria present. Errors bars represent the standard deviation of the average of three measurements in the three independent assays (n=3).

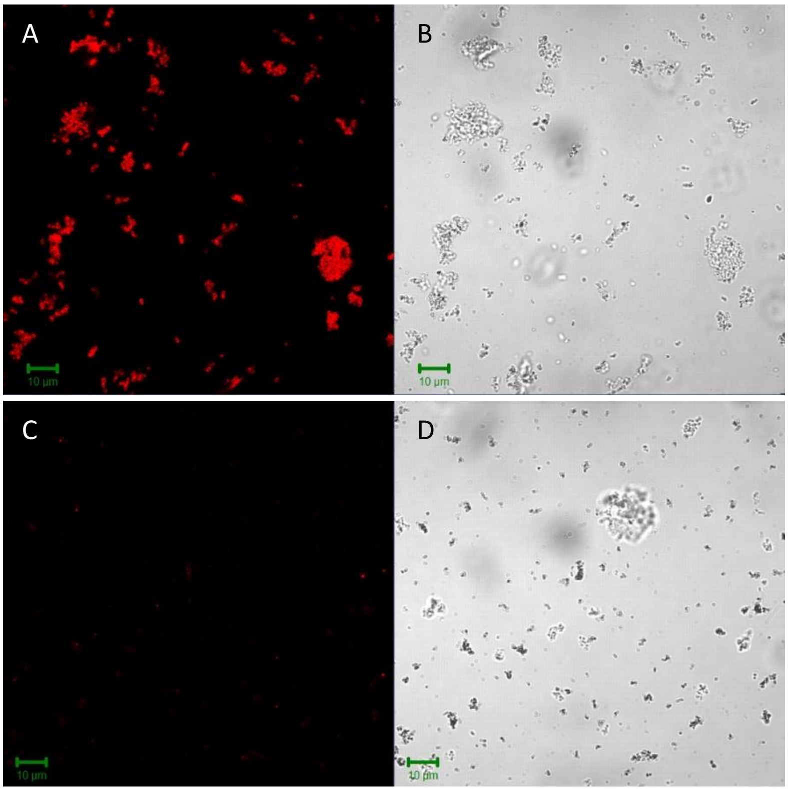

Figure S4 - Fluorescence microscopy analysis of bacterial cells after the magnetic sandwich assay performed in blood. (A-B) *E. coli* HB104 cells labeled with mCherry-Gp17 and captured on the MNPs; (C-D) *S. aureus* HB22 cells labeled with mCherry-Gp17 and captured on the MNPs.

References

Aranke, M., Moheimani, R., Phuphanich, M., Kaye, A. D., Ngo, A. L., Viswanath, O., et al. (2021). Disinfectants In Interventional Practices. *Curr. Pain Headache Rep.* 25, 21. doi:10.1007/S11916-021-00938-3.

Berney, M., Hammes, F., Bosshard, F., Weilenmann, H.-U., and Egli, T. (2007). Assessment and Interpretation of Bacterial Viability by Using the LIVE/DEAD BacLight Kit in Combination with Flow Cytometry. *Appl. Environ. Microbiol.* 73, 3283–3290. doi:10.1128/AEM.02750-06.

Boulos, L., Prévost, M., Barbeau, B., Coallier, J., and Desjardins, R. (1999). LIVE/DEAD(®) BacLight(TM): Application of a new rapid staining method for direct enumeration of viable and total bacteria in drinking water. *J. Microbiol. Methods* 37, 77–86. doi:10.1016/S0167-7012(99)00048-2.

Fernandes, E., Martins, V. C., Nóbrega, C., Carvalho, C. M., Cardoso, F. a., Cardoso, S., et al. (2014). A bacteriophage detection tool for viability assessment of Salmonella cells. *Biosens. Bioelectron.* 52, 239–246. doi:10.1016/j.bios.2013.08.053.

Gião, M. S., Wilks, S. A., Azevedo, N. F., Vieira, M. J., and Keevil, C. W. (2009). Validation of SYTO 9/propidium iodide uptake for rapid detection of viable but noncultivable Legionella pneumophila. *Microb. Ecol.* 58, 56–62. doi:10.1007/s00248-008-9472-x.

Kirchhoff, C., and Cypionka, H. (2017). Propidium ion enters viable cells with high membrane potential during live-dead staining. *J. Microbiol. Methods* 142, 79–82. doi:10.1016/J.MIMET.2017.09.011.

Liu, Y., Wang, C., Tyrrell, G., Hrudey, S. E., and Li, X. F. (2009). Induction of Escherichia coli O157:H7 into the viable but non-culturable state by chloraminated water and river water, and subsequent resuscitation. *Environ. Microbiol. Rep.* 1, 155–161. doi:10.1111/J.1758-2229.2009.00024.X.

Rosenberg, M., Azevedo, N. F., and Ivask, A. (2019). Propidium iodide staining underestimates viability of adherent bacterial cells. *Sci. Rep.* 9, 1–12. doi:10.1038/s41598-019-42906-3.

Truchado, P., Gil, M. I., Larrosa, M., and Allende, A. (2020). Detection and Quantification Methods for Viable but Non-culturable (VBNC) Cells in Process Wash Water of Fresh-Cut Produce: Industrial Validation. *Front. Microbiol.* 11. doi:10.3389/FMICB.2020.00673.

Ye, C., Lin, H., Zhang, M., Chen, S., and Yu, X. (2020). Characterization and potential mechanisms of highly antibiotic tolerant VBNC Escherichia coli induced by low level chlorination. *Sci. Rep.* 10, 1–11. doi:10.1038/s41598-020-58106-3.
